# Influence of circadian rhythm on the determination of the IMMune Age indeX (IMMAX)

**DOI:** 10.3389/fragi.2025.1716985

**Published:** 2025-11-07

**Authors:** Sina Trebing, Peter Bröde, Maren Claus, Carsten Watzl

**Affiliations:** Department for Immunology, Leibniz Research Centre for Working Environment and Human Factors (IfADo), Dortmund, Germany

**Keywords:** circadian, chronotype, immune age, immune cell, immunosenescence

## Abstract

**Introduction:**

To study the impact of immunosenescence on various biological processes the determination of the immune age has gained much interest in recent years. Based on easily determined five immune cell components we recently developed the IMMune Age indeX (IMMAX) as a biomarker for immunosenescence. However, the influence of the circadian rhythm of immune cell concentrations in peripheral blood on the determination of the IMMAX was unclear.

**Methods:**

Therefore, we conducted an experimental study with 50 participants aged 20–69 years and took blood samples at 8 a.m., 1 p.m. and 6 p.m. to determine the IMMAX over the day. Additionally, the chronotype of the study participants was assessed by D-MEQ questionnaire.

**Results:**

While individual immune cell ratios showed changes during the day, we determined no significant variability of the IMMAX score between the measurements. Interestingly, the individual chronotype influenced the diurnal rhythm of the IMMAX score with morning types showing a decrease of IMMAX values during the day.

**Conclusion:**

This longitudinal trial strengthens the IMMAX as a robust biomarker for the immune age independent from time of blood sampling.

## 1 Introduction

With aging several changes in the immune system with regards to cell counts, effector functions and soluble mediators can be observed. This process is called immunosenescence and includes a rise in memory T cells, a decrease in naïve T cells and increased systemic inflammation (Aiello et al., 2019). As a consequence, older individuals are more susceptible to chronic diseases, cancer, and infections, whereas vaccine efficacy is reduced (Aiello et al., 2019). Immunosenescence is strongly correlated with chronological age, explaining why age is a strong predictor of mortality and why most recommendations for adult vaccinations use age as a criterion. However, the velocity of immune aging can vary between individuals of the same chronological age. Determinants for accelerated or decelerated immunological ageing are sex in favor of women, ethnicity, exposure to pathogens, e.g., CMV status, and lifestyle factors such as nutrition and physical activity (Hirokawa et al., 2013; Turner, 2016; Maijo et al., 2014; Jergovic et al., 2019). Therefore, several immunological age metrics were developed over the past decade and have proven themselves to be more precise in describing immunosenescence than chronological age (Alpert et al., 2019; Bröde et al., 2022). Horvath originally developed the first algorithm for determining biological aging across organs using methylation patterns of DNA as the basis of the epigenetic clock (Horvath, 2013). Since immune cells are easily accessible through peripheral leukocytes, advanced epigenetic clocks now use the DNA methylation age of these cells. They have already demonstrated initial longitudinal clinical relevance in terms of mortality and disease risk prediction (Sabbatinelli et al., 2024). Finally, there are measurement methods based on the distribution of naïve and senescent immune cells, showing that immunological aging processes can be evaluated in various, certainly linked ways.

We recently developed a metric called IMMune Age indeX (IMMAX) which is composed of immune cell proportions. This practically feasible biomarker consists of five relative cell counts and ratios, namely, NK/T cell ratio, CD4^+^/CD8^+^ T cell ratio, memory/naïve CD4^+^ and CD8^+^ T cell ratios, and CD28^-^-CD8^+^ T cells assessed by flow cytometry (Bröde et al., 2022). It shows a high correlation with more complex omics-based measures of immune age (Alpert et al., 2019; Bröde et al., 2022). Moreover, two longitudinal aging-related studies in Finland revealed a tight association between DNA methylome age estimation and immunosenescence-associated characteristics, that are included in the IMMAX components (Kananen et al., 2016). Besides the strong correlation between IMMAX and chronological age, we could show lower values in females and that IMMAX was a better predictor for cardiorespiratory fitness than chronological age (Bröde et al., 2022). A five-year follow-up in 53 participants from a cohort study showed an average IMMAX increase of +0.02, equivalent to an additional 4.8 years of chronological age, according to Bröde’s percentile model (Bröde et al., 2023). Apparently, this biomarker appears to be useful in the long term. However, further development is essential to identify and eliminate possible bias. Not considered to date is the time of blood sampling since the immune system is a strongly circadian regulated system.

It is well established that several immune cell populations are influenced by the circadian rhythm (Dimitrov et al., 2009). On the one hand, there are cortisol-sensitive cells with high CXC motif chemokine receptor 4 (CXCR4) expression leading to a nighttime peak in peripheral blood when cortisol levels are low. These include naïve T cells, central memory, and effector memory T cells. On the other hand, natural killer (NK) cells and effector CD8^+^ T cells are controlled by catecholamines via beta-adrenergic and fractalkine receptors (CX3CR1). Consequently, catecholamine-induced demargination of these cells from endothelium causes a daytime peak and nighttime nadir. In addition to these endocrine mechanisms, trafficking of innate and adaptive immune cells is also regulated by intrinsic pathways. A sophisticated system comprising clock genes and transcription factors forms a peripheral clock which is synchronized to the light-dark cycle (Scheiermann et al., 2018; Bollinger et al., 2011). Next to the well-known master clock transcription factors Brain and muscle Arnt-like protein-1 (BMAL1) and circadian locomotor output cycles kaput (CLOCK), chemokine receptor (CCR7) and sphingosine-1-phosphate-receptor 1 (S1PR1) are circadian-controlled regulators of T cell migration (Druzd et al., 2017). CCR7 peaks in phase with chemokine ligand 21 (CCL21) expressed on high endothelial venules (Druzd et al., 2017). CCL21-mediated cell homing to secondary lymphoid organs at the beginning of the active phase underlines the microenvironment’s role in diurnal oscillations. Additionally, proinflammatory cytokine concentrations follow a circadian rhythm with a peak of TNF during the night (Lange et al., 2010).

These circadian oscillations of immune cells are also of clinical relevance in the context of proinflammatory immune response to an acute infection (Bellet et al., 2013) or vaccine efficacy. In a recently published review, Otasowie et al. coined the term ‘chronovaccination’ (Otasowie et al., 2022). Based on several randomized controlled trials (RCTs), vaccinations against influenza, hepatitis A virus and SARS-CoV-2 in the morning seem to result in higher antibody responses (Phillips et al., 2008; Zhang et al., 2021). While the exact mechanisms for this effect are not fully understood, current data indicate a crucial role of molecular clocks in immune cells, whereas no associations to systemic cortisol and steroid hormone levels were found (Long et al., 2016).

The chronotype refers to an innate preference for being physically or cognitively active at certain times. Self-reported questionnaires, such as the Morningness-Eveningness-Questionnaire (MEQ) by Horne and Ostberg (Horne and Ostberg, 1976), are commonly used to determine an individual’s chronotype. Besides proven split-half as well as test-retest reliability (Adan and Natale, 2002; Griefahn et al., 2001; Adan and Natale, 2002; Griefahn et al., 2001) and a high internal consistency of Cronbach α = 0.83 (Paine et al., 2006), MEQ scores have shown high correlations with three objective circadian phase markers, namely, body temperature, melatonin and cortisol levels (Griefahn et al., 2001; Baehr et al., 2000; Bailey and Heitkemper, 2001). Time-delayed hormone levels across different chronotypes may result in shifted rhythms of cortisol-sensitive immune cell proportions. However, only little is known about the influence of the chronotype on immune cell circadian rhythms.

As the IMMAX is based on immune cell counts and ratios in peripheral blood, we speculated that circadian fluctuations of different immune cells may impact the determination and the accuracy of IMMAX when blood sampling is performed at different timepoints during the day. Additionally, this may further be influenced by the individual chronotype. Therefore, we performed a longitudinal experimental study measuring the IMMAX for 50 study participants aged 20–69 years in the morning, at noon and in the evening of the same day.

## 2 Materials and methods

### 2.1 Study design

50 participants were recruited between December 2023 and April 2024. In order to obtain a wide range of IMMAX values, we recruited at least 15 participants for each of three age categories (18-35/35-55/>55 years old). Individuals with chronic-inflammatory or malignant diseases, medication with potential influence on immune cell count or function, and present participation in other drug treatment studies were excluded. Additional exclusion criteria were <10 days since last infection or vaccination with an inactivated vaccine or <4 weeks since vaccination with attenuated live vaccine. The participants were asked not to do any moderate or intensive physical activity on the examination day. The study involved three measurement timepoints (t_08_ at 8 a.m., t_13_ at 1 p.m., t_18_ at 6 p.m.) at which 2.7 mL blood was drawn from a cubital vein. Additionally, demographic, anamnestic and anthropometric data was collected at t_08_. The D-MEQ, German version of the Morningness-Eveningness-Questionnaire, was fulfilled to determine the chronotype (Griefahn et al., 2001). It consists of 19 questions about preferred timeframes for work, activities and sleep. The sum score ranges from 14 to 86 points, resulting in five different categories (definite evening type (14-30), moderate evening type (31-41), neutral type (42-58), moderate morning type (59-69) and definite morning type (70-86). The study was approved by the ethics committee of the Leibniz Research Centre for Working Environment and Human Factors (#243).

### 2.2 IMMAX calculation procedure

100 μL of the EDTA-anticoagulated blood was directly immunostained with a combination of seven antibodies (BV421 anti-CD4 (clone RPA-T4, BD Horizon™), BV510 anti-CD3 (clone UCHT1, BD Horizon™), BB515 anti-CD8 (clone RPA-T8, BD Horizon™), PerCP-Cy™ 5.5 anti-CD28 (clone CD28.2, BD Pharmingen™), PE anti-CCR7 (CD197) (clone 3D12, BD Horizon™), PE-Cy™5 anti-CD56 (clone B159, BD Pharmingen™), Alexa Fluor® 700 anti-CD45RA, (clone HI100, BD Pharmingen™) and a Fixable Viability Dye eFluor™ 780 in Brilliant Stain Buffer (BD Biosciences, San Jose, California, United States). After incubation for 30 min at room temperature, red blood cells were lysed with 1 mL of FACS Lysing Solution (BD Biosciences). Samples were centrifuged, supernatant was discarded, and pellets were resuspended in 250 μL of flowcytometry buffer (PBS +2% FCS). 30.000 lymphocytes were acquired by FACS DIVA Software using BD LSRFortessa™ Cell Analyzer. Data were analyzed using FlowJo™ v10.10 Software (BD Life Sciences). Gating strategy as published recently (Bröde et al., 2023) was used to determine the following relative cell frequencies: NK/T cell ratio, CD4^+^/CD8^+^ T cell ratio, memory/naïve CD4^+^ and CD8^+^ T cell ratios, CD28^-^-CD8^+^ T cells. IMMAX values were calculated from the relative cell frequencies as described before and converted into equivalent years of life (EYOL) and an age gap (= EYOL based on the IMMAX–chronological age) (Bröde et al., 2023).

### 2.3 Statistical analysis

Statistical analysis was performed using GraphPad Prism 10 version 10.3.0 for macOS X (Boston, Massachusetts, United States) and R Statistical Software (v4.5.1 R Core Team 2025). Chi-Square-test and Kruskal-Wallis-test were used to assess baseline differences across the three age categories regarding demographic, anthropometric data and chronotype. Correlation analysis of the total population’s (n = 50) chronological and immunological age described as median EYOL was executed to validate the IMMAX data. Beyond that, unpaired t-test was applied to detect sex differences concerning the median age gap. Pairwise comparisons with paired t-tests were performed to evaluate diurnal trends of the IMMAX and its single components between t_08_, t_13_ and t_18_. P-values were adjusted for multiple testing. For evaluating the diurnal trend, the baseline-adjusted Area Under the Curve (AUC_netto_) was calculated after subtracting IMMAX at t_08_ from all three IMMAX values at t_08_, t_13_ and t_18_. Subsequently, multiple linear regression analysis of the AUC_netto_ and predicted by chronotype, age, BMI, and sex was calculated. In addition, subgroup analysis concerning the effect of the three chronotypes (morning, neutral, evening type) on IMMAX was performed. Level of significance (α) was set to 0.05.

## 3 Results

Of the 50 participants recruited, 36 were of normal weight, 10 were overweight (BMI 25-29,9 kg/m^2^), and 4 were obese (BMI >30 kg/m^2^). The chronotype classification resulted in 19 morning, 23 neutral and 8 evening types according to the D-MEQ questionnaire. The characteristics of the participants are shown in Table 1, with no significant group differences in sex, height, weight, BMI and D-MEQ score.

**TABLE 1:** Descriptive characteristics of the study participants grouped by age category.

Parameter	Age category I (18-24 years) n = 17			Age category II (35-54 years) n = 17			Age category III (>54 years) n = 16		
sex (F/M)	9/8			9/8			11/5		
	Min	Max	Mean (SD)	Min	Max	Mean (SD)	Min	Max	Mean (SD)
age (years)	20	34	28.29 (3.80)	35	54	42.94 (6.61)	55	69	62.81 (4.88)
height (cm)	167	196	178.7 (9.01)	162	189	172.9 (8.42)	157	195	170.80 (10.60)
weight (kg)	55	95	73.74 (12.68)	53	115	72.88 (15.52)	53	120	71.88 (19.28)
BMI (kg/m^2^)	18.87	28.70	22.96 (2.59)	19.16	32.19	24.23 (3.80)	19.95	34.32	24.32 (4.11)
D-MEQ score (points)	37	74	52.18 (9.70)	37	75	58.53 (11.02)	34	79	53.50 (12.36)

**FIGURE 1:**
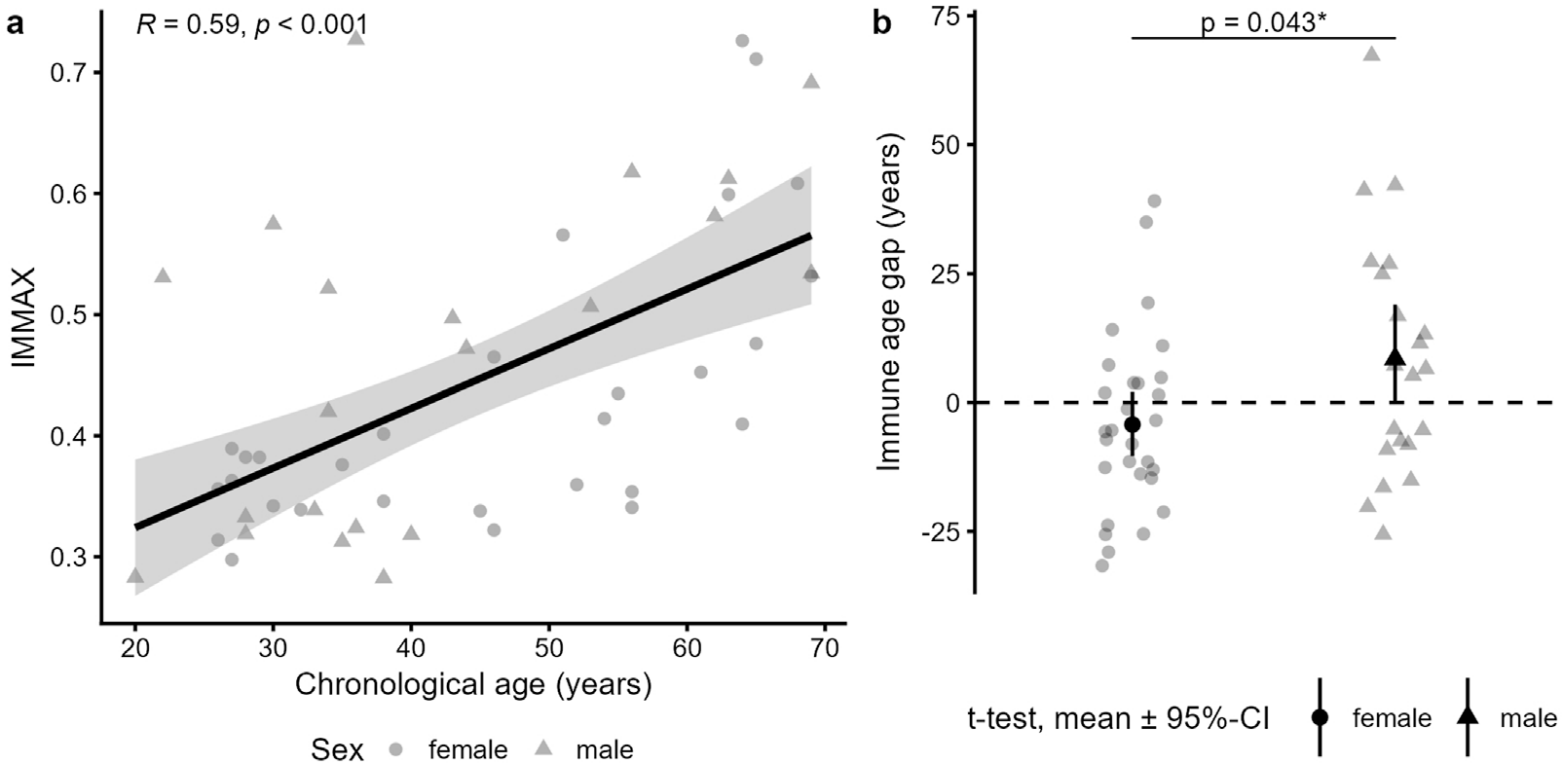
IMMAX and age gap description of the study participants. **(a)** Pearson correlation of the subjects’ (n = 50) chronological age und IMMAX (calculated as average IMMAX of the three time points t_08_, t_13_ and t_18_). **(b)** Immune age gap in female (n = 29) versus male (n = 21) subjects, tested by t-test.

Confirming previous results (Bröde et al., 2022), the IMMAX score showed a moderately strong correlation with the participants’ chronological age (r = 0.59, p < 0.001) (Figure 1a). While the average age gap was almost zero (mean age gap = 1.09 years (SD ± 20.93)), women (n = 29) had a significantly lower mean age gap of −4.25 years (SD ± 17.32) compared to men (n = 21) with a mean age gap of +8.46 years (SD ± 23.56; p = 0.043) (Figure 1b). This confirms previous results showing that women are immunologically about 1 decade younger than men (Bröde et al., 2022; Alpert et al., 2019).

**FIGURE 2:**
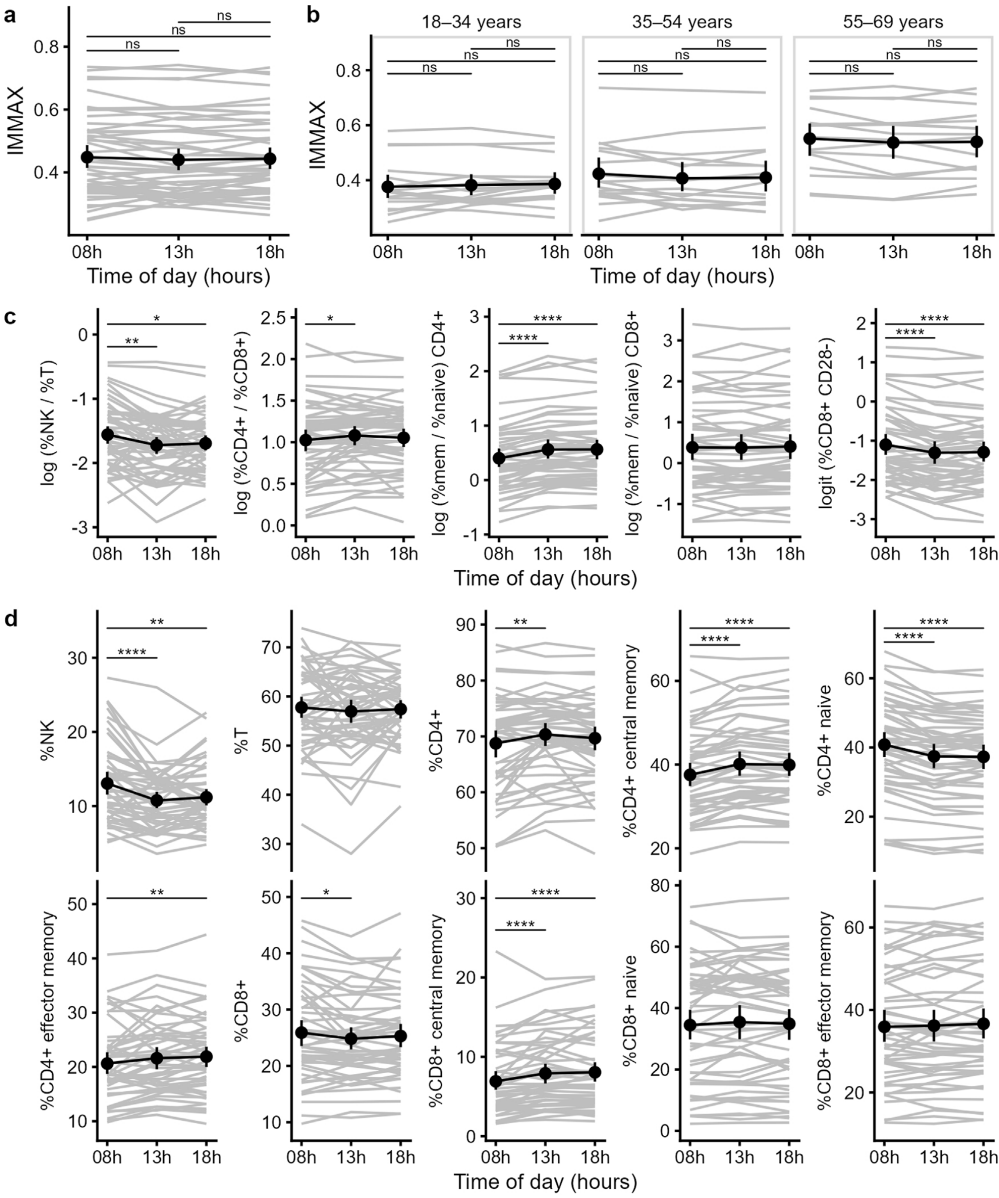
Diurnal trend of the IMMAX and its components. **(a,b)** Individual and mean IMMAX values from 8 a.m. to 6 p.m. of the total sample **(a)** or for the three different age categories **(b)**. **(c)** Trends of the five IMMAX components, using log-transformed cell ratios and logit transformed CD28^-^-CD8^+^ T cell percentages. **(d)** Values for the individual markers used to calculate the five IMMAX components over the three measuring time points. **(a–d)** Means and 95%-CI, P-values of pairwise comparisons with paired t-test adjusted for multiple testing; *P < 0.05, **P < 0.01, ***P < 0.001, ****P < 0.0001.

After validating our IMMAX scores with known results, we compared the individual IMMAX values in the morning, at noon and in the evening. We found no significant difference between the IMMAX values during the day, neither for the whole sample (Figure 2a) nor for a specific age category (Figure 2b). Concerning the individual IMMAX components, we found significant differences between the morning sample and the later hours (Figure 2c). The NK/T ratio was slightly elevated at the first measuring time point, which was mostly caused by higher frequencies of NK cells at the first time point (Figure 2d). Similarly, the percentage of CD28^-^-CD8^+^ T cells decreased from the first to the following measurements. In contrast, the memory/naïve CD4^+^ ratio was slightly higher at the noon and evening measurement, which was due to an increase of central and effector memory CD4 T cells and a drop of naïve CD4 T cells during the day. Also, the CD4/CD8 ratio showed a slight increase between the morning and the noon time point due to an increase of CD4 and a drop of CD8 T cells. Only the memory/naïve CD8^+^ ratio was stable during the day as we only observed a slight increase in CD8 central memory T cells during the day. Therefore, while individual IMMAX components showed some changes between the morning and the later measuring time points, these were opposing effects (decreased NK/T ratio and CD28^-^-CD8^+^ T cells and increased memory/naïve CD4^+^ ratio and CD4/CD8 ratio), which balanced the IMMAX during the day.

**FIGURE 3:**
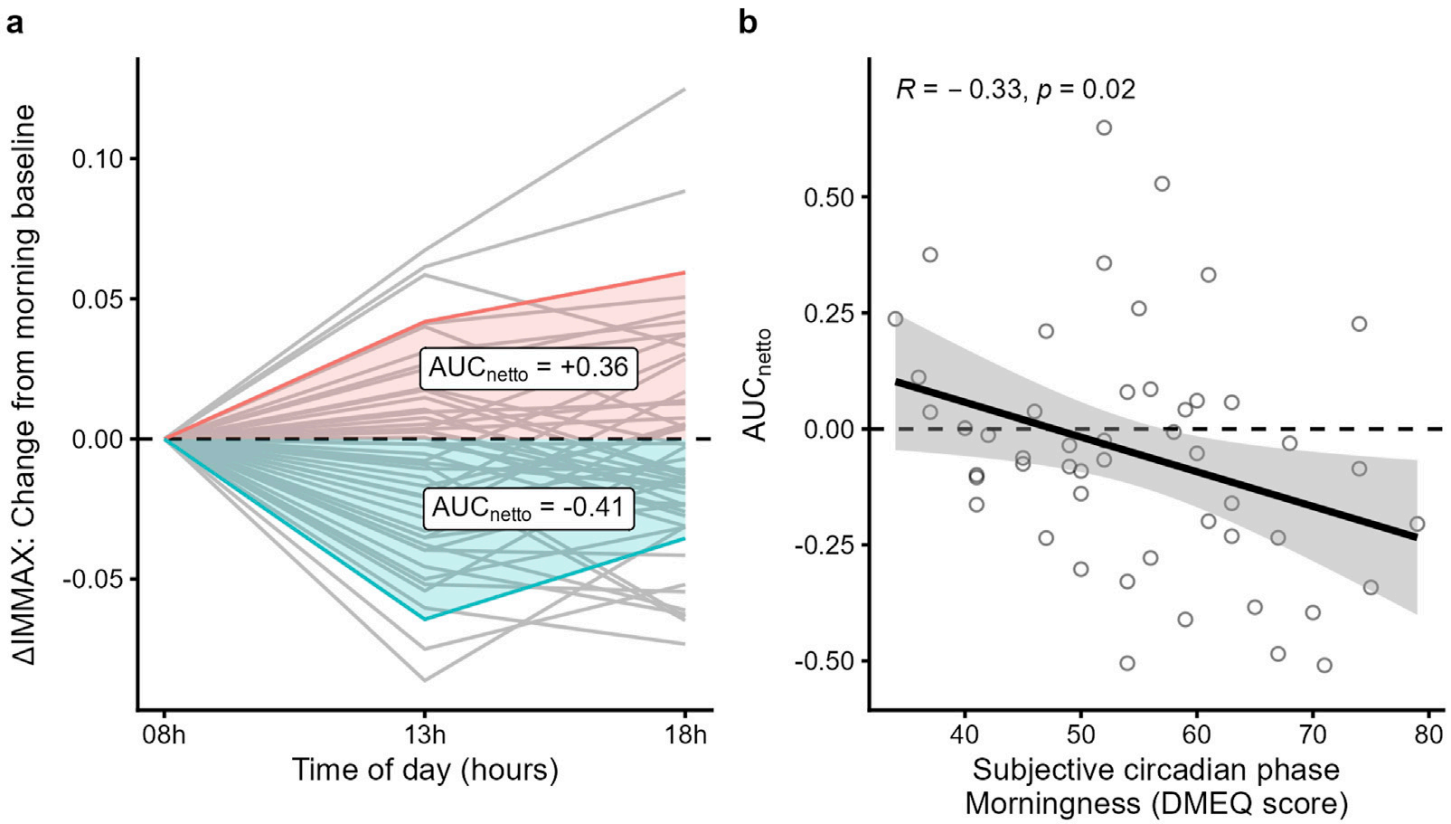
Diurnal IMMAX changes correlate with the D-MEQ score. **(a)** AUC_netto_ calculation of the IMMAX differences using IMMAX_t08_ as baseline; two example curves, one positive, colored red, and one negative, colored blue, are highlighted. **(b)** D-MEQ score (range: 34-79 points) and morning baseline corrected AUC_netto_ linear regression analysis with 95%-CI and Pearson correlation coefficient.

When analyzing the results of the D-MEQ score we found no significant correlation with the IMMAX or the age gap of the study participants. While the IMMAX was generally stable during the day (Figure 2a), we noticed considerable interindividual variability of the IMMAX levels. Figure 3a shows the values after subtracting the 8 a.m. IMMAX value as base line and plotted over time. The netto area under the curve (AUC_netto_) calculated from these values quantify the diurnal trend, where positive values represent an increase and negative values a decrease of the IMMAX during the day (Figure 3a). When comparing the AUC_netto_ with the D-MEQ score we found a significant negative correlation (r = −0.33, p = 0.02) (Figure 3b). The higher the D-MEQ score, corresponding to a morning type tendency, the more likely the IMMAX decreases from morning to midday and evening. Beyond the univariate analysis, this effect was confirmed by the multiple regression analysis, whereas age, BMI (normal vs. overweight/obese), and sex (female vs. male) were not found to be significant factors (Supplementary Figure S1).

## 4 Discussion

The use of the IMMAX as a simple measure of immune age in several study settings raised the methodological question of its intra-day stability. Here, the correlation between chronological age and the IMMAX confirms that the IMMAX is a plausible construct of immune age. Furthermore, the study population shows an IMMAX gender gap of a similar magnitude to the large Dortmund Vital Study sample (Bröde et al., 2022) of over 500 participants, considered a representative sample for the regional working population (Gajewski et al., 2022). The statistical analysis revealed no time-of-day effect on the IMMAX score in the overall study cohort, suggesting that the time of blood sampling has no major impact on the IMMAX score in a large study population. However, when looking at intraindividual results, the IMMAX varied in median by ± 0.035 throughout the day (Figure 3a). Given that the IMMAX ranges from 0 to 1, this variability is not particularly impressive and is, as proven statistically here, negligible in a larger population. When converted to equivalent years of life, which is applied to make the IMMAX more tangible, this represents a variance of ±7 years. If individuals’ values are communicated as EYOL, 7 years are perceived as a significant amount. Therefore, the factors influencing this variance were explored, and it was found that chronotype was relevant, with pronounced changes throughout the day for the morning type.

Morning types, often called *larks* in common parlance, tend to have decreasing IMMAX values throughout the day. The D-MEQ is a self-reported questionnaire, but it has shown a good correlation with dim light melatonin onset, which is the diagnostic gold standard of an individual’s endogenous circadian phase (Griefahn et al., 2001). Within the first hour of waking, Kudielka et al. (2006) found that the amplitude of salivary cortisol was higher in morning types than in evening types, as assessed by the MEQ (Kudielka et al., 2006; Horne and Ostberg, 1976). A single day study by Petrowski et al. (2020) supported these findings with a significantly elevated cortisol level upon awakening in morning types (Petrowski et al., 2020). These results may explain the distinct diurnal trend as a consequence of higher cortisol-mediated immune cell shifts in *larks* as outlined below.

Given the first measurement time point at 8 a.m. and an earlier, more pronounced cortisol peak in morning types (Kudielka et al., 2006), the negative correlation between the netto AUC and D-MEQ score (Figure 3b) could possibly be attributed to an earlier T cell decline due to cortisol secretion and CXCR4 activation (Dimitrov et al., 2009). In addition, the catecholamine-induced diurnal peak (Dimitrov et al., 2009) has already started in morning types, leading to higher NK cell values. Both hormone-induced shifts boost the NK/T cell ratio as an important IMMAX component. This may explain the negative netto AUC, indicating a decreasing IMMAX within the following 5 (t_13_) or 10 (t_18_) hours. Indeed, a chronotype-related subgroup analysis revealed that the overall diurnal trend of a decreasing NK/T ratio as one of the five IMMAX components (Figure 2c) was based exclusively on the 19 morning types. When only the neutral and evening types were analyzed, this trend was no longer confirmed.

The IMMAX as an immunologic age marker is based on cell ratios without knowledge of absolute cell counts. Lange et al. (Lange et al., 2010) described cell count trends in 14 male subjects at five time points during the day (8, 11, 14, 17, 20 h) which may appear contrary to our findings: In contrast to the decrease in NK cells from morning to noon, they found an increase in absolute NK cell numbers. However, a supplementary analysis of absolute cell counts in a small subgroup of three morning type study participants indicated that the significant decline in the NK/T cell ratio is caused by both an absolute decrease in NK cell counts and an increase in T cells (Supplementary Figure S2). The first blood sampling may have pushed the NK cells up by adrenergic stimulation in some participants and this response may have been attenuated during the course of the day.

Our study has certain limitations. T_08_ was defined by the time of day in our protocol and was not synchronized with the participants’ wake-up time. Hormonal changes around awakening that induce the switch from resting to active phase, such as the cortisol awakening response, may have an impact on the IMMAX-determining immune cell subpopulations which showed significant differences between the first measurement time point at 8 a.m. and the subsequent time points (t_13_, t_18_). The lack of synchronization may have led to a certain amount of noise in the measurements of immune cell populations. Further limitations include that we did not ask about sleep on the previous night or current stress level. Lange et al. demonstrated that regular sleep facilitates T cell proliferation and NK cell counts in the following afternoon and evening, in contrast to continuous wakefulness (Lange et al., 2010; Born et al., 1997). In addition, cortisol and catecholamine secretion are altered by stress and sleep disturbance.

Finally, the term ‘chronovaccination’ refers to vaccine responses at different time points during the day (Otasowie et al., 2022). Vaccine efficacy is influenced by several immune cell populations, mainly B cells, dendritic cells, and T helper cells. Naïve B cell counts in the blood can predict the humoral response to SARS-CoV-2 mRNA vaccination in immunocompromised patients (Schulz et al., 2021). However, B cells were not analyzed in the dataset used for initial establishment of the IMMAX (Bröde et al., 2022), meaning that these cells are not included in the IMMAX components. The same applies to dendritic cells. Their migration to lymph nodes shows circadian migration patterns (Holtkamp et al., 2021), which is also likely to have an important impact on vaccine antigen trafficking. By only considering T helper cells that enhance B cell activation in the IMMAX, the relevance of our results to chronovaccination may be limited. Nonetheless, data recently published by Davies et al. (2025) and using the IMMAX tool shows that immune age is negatively correlated with antibody responsivity to SARS-CoV-2 vaccination in older individuals. This suggests that the IMMAX at least indirectly also correlates with B cell aging, providing an indication of the comprehensive immunosenescence process. In addition, chronotypes have not yet been taken into account in time-dependent vaccination studies. Shifted and pronounced rhythms in morning types, as shown here, suggest certain differences and could advance this field of research towards individualized infection prevention strategies.

## 5 Conclusion

In conclusion, the IMMAX calculation in larger populations can be performed at any time of day. However, for further research projects, it is recommended to use a defined time point and a minimum time interval from awakening for blood collection when measuring subjects with different chronotypes, BMI, and gender in order to increase accuracy.

## Data Availability

The raw data supporting the conclusions of this article will be made available by the authors, without undue reservation.

## Abbreviations

AUC, area under the curve; BMI, body mass index; BMAL1, Brain and muscle Arnt-like protein-1; CCR7, C-C chemokine receptor type 7; CI, confidence interval; CLOCK, circadian locomotor output cycles kaput; CMV, cytomegalie virus; CXCR4, CXC motif chemokine receptor 4; CX_3_CR1, motif chemokine receptor 1, fractalkine receptor; D-MEQ, German version of the Morningness-Eveningness-Questionnaire; EDTA, Ethylenediaminetetraacetic acid; EYOL, equivalent years of life; IMMAX, Immune Age Index; MEQ, Morningness-Eveningness-Questionnaire; NK cell, natural killer cell; RCT, randomized controlled trial; SARS-CoV-2, severe acute respiratory syndrome coronavirus type 2; S1PR1, sphingosine-1-phosphate-receptor 1; TNF-α, tumor necrosis factor alpha.
