# Correction: Influence of circadian rhythm on the determination of the IMMune Age indeX (IMMAX)

**DOI:** 10.3389/fragi.2025.1743195

**Published:** 2025-11-18

**Authors:** Sina Trebing, Peter Bröde, Maren Claus, Carsten Watzl

**Affiliations:** Department for Immunology, Leibniz Research Centre for Working Environment and Human Factors (IfADo), Dortmund, Germany

**Keywords:** circadian, chronotype, immune age, immune cell, immunosenescence

The title of this article was erroneously given as “Influence of circadian rhythm on the determination of the IMMune age indeX (IMMAX).” The correct title of the article is “Influence of circadian rhythm on the determination of the IMMune Age indeX (IMMAX).”

The original article has been updated.

