# Supplementary material for: Influence of circadian rhythm on the determination of the IMMune Age indeX (IMMAX)
Source: Front Aging. 2025 Nov 7;6:1716985. doi: 10.3389/fragi.2025.1716985 (PMC12671377; doi:10.3389/fragi.2025.1716985)
Supplement: Supplementary file 1 [file Supplementaryfile1.docx]

Supplementary Material

## Supplementary Figures


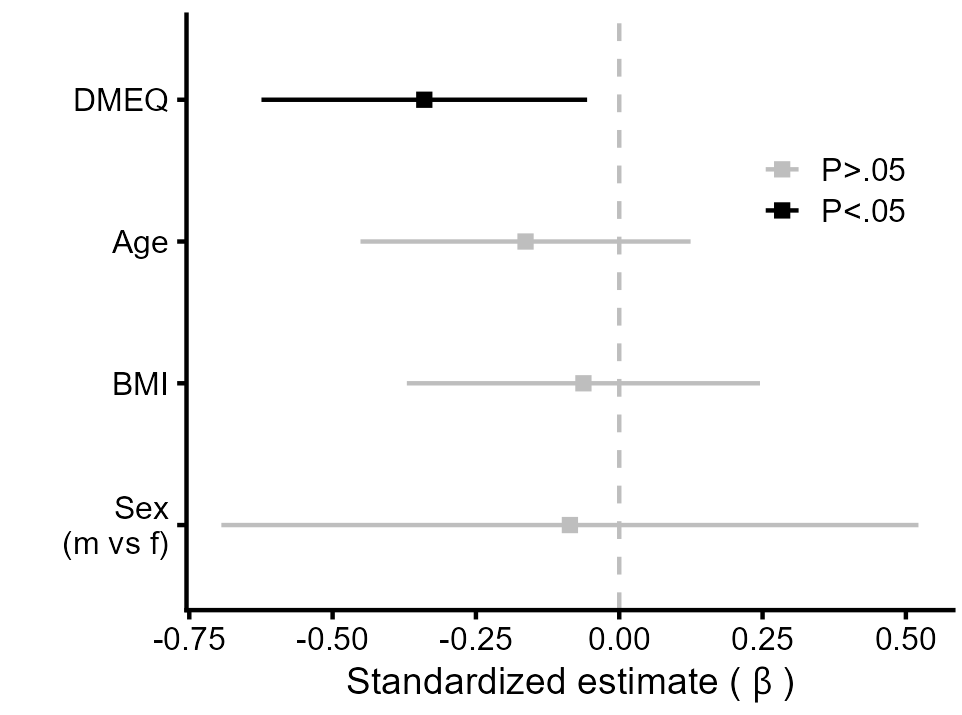


**Supplementary Figure 1. Multiple regression analysis of D-MEQ, age, BMI and sex as factors predicting diurnal IMMAX trends.** Standardized coefficients with 95% confidence intervals from a multiple regression predicting the diurnal trend (AUC_netto_) of the IMMAX by chronotype (DMEQ), age, body-mass-index (BMI) and sex (male versus female). The significant effect of DMEQ is shown in black (p=0.020) and the non-significant factors (p>0.05) with the 95% CI overlapping with the vertical reference indicating null effect are displayed in grey.


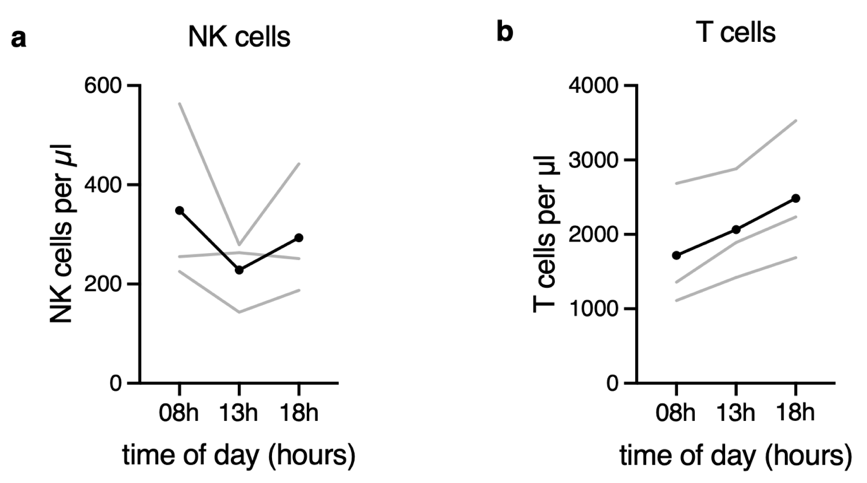


**Supplementary Figure 2.** **Absolute numbers of NK cells and T cells in a small subgroup of morning type study participants.** Absolute concentrations of NK cells (a) and T cells (b) at 8 a.m., 1 p.m., and 6 p.m. of three study subjects classified as morning types (*gray curves*) and their mean (*black curve*). 50 μL of EDTA-anticoagulated blood was reverse pipetted into BD Trucount^TM^ Tubes (Cat. No. 663028) and stained with 0.5 μL each of BV421 anti-CD19, PerCP-Cy5.5 anti-CD56, BV510 anti-CD3, PE anti-CD14, AF700 anti-CD45. After 20 minutes incubation 500 μL BD FACS^TM^ Lysing Solution (Cat. No. 349202) at 10% dH_2_O dilution was added. The cells were again incubated for 10 minutes again before analysis on a LSRFortessa flow cytometer. The absolute number of positive cells was then calculated using the following formula: *( #events in cell population / #events in absolute count bead region) x ( #beads/test / test volume (50 μL)) = absolute cell population count.*
